# 227 Implementation of a Penicillin Allergy De-labeling Program in a Veterans Affairs Community Living Center

**DOI:** 10.1017/ash.2026.10606

**Published:** 2026-06-23

**Authors:** Sarah Petersen, Daniel Muleta, Christopher Evans, Cherlly Bailey, Carissa Wallace, Becky Meyer

**Affiliations:** 1 Tennessee Department of Health; 2 TN Department of Health; 3 TN Dept of Health; 4 TENNEESE DEPARTMENT OF HEALTH

## Abstract

**Background:** Extended-spectrum beta-lactamase-producing Enterobacterales (ESBL-E) are a growing public health concern. This study investigates antibiotic resistance patterns for ESBL-E (Escherichia coli and Klebsiella species) cases in Maury and surrounding counties in Tennessee as part of the Emerging Infection Program’s Multi-site Gram-negative Surveillance Initiative. **Methods:** ESBL-E cases are defined as extended-spectrum cephalosporin-resistant E. coli, Klebsiella pneumoniae, Klebsiella variicola, or Klebsiella oxytoca isolated from urine or normally sterile sites from residents of the surveillance area. Cases from July 2019 through December 2024 were included. Susceptibility data were collected from clinical laboratory reports. E. coli isolate resistance was compared to aggregated Klebsiella species isolate resistance using χ or fisher’s exact tests. Organism specific yearly resistance trends excluded the incomplete 2019 collection year and were examined via simple linear regression. Analyses were performed in SAS 9.4. **Results:** We identified 2,206 isolates including 1,915 E. coli isolates, of which 1,868 (97.5%) were from urine, and 291 Klebsiella isolates, of which 281 (96.6%) were from urine. Overall percent resistance for selected antibiotics were as follows: ciprofloxacin: 69.0%, levofloxacin: 64.3%, cefepime: 81.1%, cefazolin: 98.7%, cefotaxime: 85.9%, ceftazidime: 53.2%, ceftriaxone: 92.8%, piperacillin-tazobactam: 2.6%, nitrofurantoin: 9.0%, trimethoprim-sulfamethoxazole: 57.8%. During the study period, E. coli isolates displayed increased resistance to cefepime (3.0% per year, P<0.001), ceftriaxone (1.3% per year, P:P: 0.02); Klebsiella isolates demonstrated increased resistance to levofloxacin (5.9% per year, P: 0.002) and cefepime (5.7% per year, P: 0.003). Over the study period, E. coli isolates displayed higher rates of resistance than Klebsiella isolates to ciprofloxacin (74.0% vs. 36.4%, P<0.001), levofloxacin (71.4% vs. 19.0%, P<0.001), cefotaxime (87.0% vs. 79.0%, P<0.001) and ceftriaxone (93.5% vs. 87.9%, P<0.001). Klebsiella isolates had higher resistance to ceftazidime (59.9% vs. 52.1%, P: 0.01), piperacillin-tazobactam (8.9% vs. 1.6%, P<0.001), nitrofurantoin (18.6% vs. 7.6%, P<0.001), and trimethoprim-sulfamethoxazole (68.6% vs. 56.1%, P<0.001). There were no significant differences in resistance to cefepime or cefazolin between groups. **Conclusion:** In our data, ESBL-E pathogens demonstrated trends of increasing resistance to certain antibiotics over time. There were significant differences in resistance between species within different classes of antibiotics. These data support the idea that organism type is important for determining the best antibiotic for empiric treatment. Continued monitoring of antibiotic resistance in ESBL-E is imperative to understanding changes in antibiotic resistance patterns. The increasing trends in proportion of antimicrobial resistant strains warrants strengthening antimicrobial stewardship and infection prevention programs within the surveillance area.

